# Editorial: Redefining acute psychiatric care: strategies for improved inpatient experiences

**DOI:** 10.3389/fpsyt.2025.1775691

**Published:** 2026-01-13

**Authors:** Alfonso Ceccherini-Nelli, Joseph O. Fadare, Stefano Barlati

**Affiliations:** 1 Department of Psychiatry, University of Alberta, Edmonton, AB, Canada; 2 Alberta Health Care Services, Edmonton, AB, Canada; 3 Department of Medicine, Ekiti State University Teaching Hospital, Ado Ekiti, Nigeria; 4 Department of Pharmacology and Therapeutics, College of Medicine, Ekiti State University, Ado-Ekiti, Nigeria; 5 Department of Clinical and Experimental Sciences, University of Brescia, Brescia, Italy; 6 Department of Mental Health and Addiction Services, ASST Spedali Civili of Brescia, Brescia, Italy

**Keywords:** acute day units, acute psychiatric care, emergency departments, inpatient psychiatric wards, mental health crises

Although there has been a shift toward community-based psychiatric care over the past six decades, hospitalization remains necessary for many individuals with severe mental illness. As a result, inpatient psychiatric services continue to demand greater economic resources than community-based alternatives, regardless of a country’s level of economic development (1).

In this Research Topic, Iwanaga et al. employed a random forest model to identify key predictors for admission to high-acuity psychiatric units among 2,064 emergency inpatients in Japan. The most significant factors were clinical profile items—such as the necessity of inpatient treatment, the need for 24-hour care, symptom severity, specialized equipment, and medication management—rather than age or diagnosis.

Barruel et al. analyzed 8,870 psychiatric hospital stays in Paris to determine predictors of extended length of stay (LOS). Factors associated with LOS exceeding 30 days included age over 55, admission from outside the sector, emergency unit admission, schizophrenia, mandatory care, seclusion/restraint, somatic comorbidity in women, and treatment resistance. Protective factors included being in a relationship, travel-related episodes, and personality disorders. Understanding these predictors can help optimize care and resource allocation.

Moscovici et al. evaluated the Dynamic Appraisal of Situational Aggression (DASA) tool for predicting violence risk in mental health inpatient settings. Analyzing 3,819 admissions and 88,124 DASA scores, the researchers found that DASA scores were moderately predictive of violent incidents (AUC 0.73), with similar accuracy across forensic, schizophrenia, and acute care units, but lower accuracy in geriatric units. New risk categories were proposed to improve specificity. These findings support DASA’s utility for identifying high-risk patients and guiding interventions.

Patients may also experience abusive behaviors. In a cross-sectional survey of 203 nurses in Japanese psychiatric hospitals, Matoba et al. found that 87.1% reported at least one abusive behavior toward patients, most commonly ignoring or rejecting them. Workplace violence and recovery-oriented attitudes increased the risk of abuse, while higher moral sensitivity and longer experience reduced it. The study highlights the need for targeted interventions to prevent nurse-perpetrated abuse.

Another notable study by Nir et al. tested intranasal oxytocin (OT) administration to clinicians in a psychiatric emergency room. Patients reported deeper, more meaningful sessions and lower distress after clinician OT administration, although perceived empathy was not significantly higher. The study suggests that OT may enhance therapeutic encounters and reduce patient distress, but the results are preliminary due to the small sample size.

Insomnia, skin complications, tobacco smoking, weight gain, and other medical issues require special clinical attention in the hospital setting. Mao et al. found that 79.6% of mental health patients in Alberta, Canada, experienced sleep disturbances before discharge from psychiatric units. Key risk factors included being married or partnered, having depression, likely anxiety, and poor wellbeing. The findings highlight the need for targeted interventions, such as cognitive-behavioral therapy and psychoeducation, to address sleep issues during the transition from hospital to community care, aiming to improve recovery and quality of life.

Luo et al. found that among people with schizophrenia, chronotype (morning/evening preference) directly affects social functioning. Evening chronotypes tend to have poorer sleep quality and higher anxiety, which further impairs social functioning. Anxiety is a key mediator, while sleep quality contributes indirectly via anxiety. The findings suggest that interventions targeting sleep and anxiety may help improve social outcomes for patients with schizophrenia, especially those with evening chronotypes.

In a real-world study, Zhang et al. evaluated the effectiveness of Failure Mode and Effects Analysis (FMEA) in managing skin complications among ICU psychiatric patients. FMEA-based interventions significantly reduced skin complications from 7.56% to 3.59%. Key risk factors identified included positioning management, skin hygiene, nutritional support, ADL dependency, hypoalbuminemia, diabetes, and ICU length of stay. The study concludes that FMEA is effective for improving skin care and reducing complications in psychiatric ICU patients through early risk identification and targeted interventions.

Zheng et al. surveyed 738 patients with severe mental illness in China, finding a smoking prevalence of 52.03%, much higher than in the general population. Key risk factors included male gender, Han ethnicity, poor economic status, family smoking history, outpatient status, family history of mental illness, drug exposure, and alcohol use. Patients with schizophrenia and bipolar disorder showed higher nicotine dependence. The findings highlight the need for targeted smoking cessation interventions for this vulnerable group.

Carney et al. tested Motiv8, a nine-week multidisciplinary weight management program for adults with severe mental illness in forensic psychiatric inpatient services. The trial found Motiv8 to be feasible and acceptable, with high recruitment, retention, and satisfaction rates. While not powered to detect clinical differences, the results support a larger trial to assess effectiveness and cost-efficiency in reducing physical health inequalities for this population.

In addition, Miola et al. used machine learning to predict medical hospitalizations in bipolar disorder (BD) patients after psychiatric discharge. Among 71 BD patients, those with cardiovascular, neurological, or osteo-muscular diseases were at highest risk for future medical admissions. Models using medical data were more accurate than those using psychiatric data alone. The results emphasize the importance of monitoring and managing physical health in BD patients to reduce hospitalizations. Larger, prospective studies are needed to confirm these findings and improve preventive care for this vulnerable population.

Overall, this Research Topic has addressed several issues relating to inpatient mental health care. Findings from papers included in the Research Topic, if incorporated into practice, will contribute to improving overall patient care.
